# Supplementary material for: Erythrocyte Glutathione S-Transferase Activity as a Sensitive Marker of Kidney Function Impairment in Children with IgA Vasculitis
Source: Int J Mol Sci. 2024 Mar 28;25(7):3795. doi: 10.3390/ijms25073795 (PMC11011981; doi:10.3390/ijms25073795)
Supplement: Supplementary file 1 [file ijms-25-03795-s001.zip › ijms-2877297-supplementary.pdf]

Table S1. Routine laboratory findings in patients 3 months since the disease onset (Mann–Whitney U test)

	group	N	Min.	Max.	Centile			P
					25.	Median	75.	
ESR (mm/h)	IgAVwN	55	2.00	51.00	4.00	6.00	12.00	0.795
	IgAVN	42	2.00	38.00	4.00	7.00	14.00	
CRP (mg/L)	IgAVwN	55	0.10	19.40	0.30	0.30	1.70	0.590
	IgAVN	42	0.10	21.20	0.30	0.30	0.95	
Hb (g/L)	IgAVwN	55	108.00	149.00	124.00	132.00	136.00	0.936
	IgAVN	42	112.00	161.00	122.75	129.50	139.00	
L (x 10^9/L)	IgAVwN	55	4.80	19.40	6.70	8.10	10.10	0.790
	IgAVN	42	4.78	17.32	6.70	8.00	9.60	
Plt (x 10^9/L)	IgAVwN	55	166.00	481.00	274.00	311.00	378.00	0.154
	IgAVN	42	240.00	461.00	293.75	335.50	370.50	
BUN (mmol/L)	IgAVwN	55	1.80	7.70	3.50	3.90	4.70	0.922
	IgAVN	42	1.60	6.80	3.08	4.20	4.73	
Creatinin (mmol/L)	IgAVwN	55	27.00	81.00	32.00	39.00	46.00	0.003
	IgAVN	42	27.00	82.00	40.00	44.50	53.25	
Tot. proteins (g/L)	IgAVwN	55	60.00	78.00	67.00	70.00	73.00	0.163
	IgAVN	42	58.00	75.00	65.00	69.00	73.00	
IgA (g/L)	IgAVwN	55	0.58	3.17	1.21	1.52	1.90	0.141
	IgAVN	42	0.82	4.48	1.21	1.68	2.13	
IgM (g/L)	IgAVwN	55	0.34	1.83	0.75	0.90	1.10	0.382
	IgAVN	42	0.41	1.73	0.72	1.00	1.19	
IgG (g/L)	IgAVwN	55	5.92	17.27	8.33	9.37	10.64	0.779
	IgAVN	42	1.91	13.62	7.69	9.08	11.28	
E/mm^3	IgAVwN	55	0.00	5.00	0.00	0.00	0.00	<0.001
	IgAVN	42	0.00	4250.00	0.00	6.50	17.25	
24h protein ex.	IgAVwN	55	0.02	0.18	0.05	0.07	0.09	0.052
	IgAVN	42	0.02	0.94	0.05	0.08	0.11	
eGFR (ml/min/1,73m^2)	IgAVwN	55	81.58	156.48	97.36	114.60	133.45	0.001
	IgAVN	42	74.36	142.22	89.40	101.05	115.05	

Legend: IgAVwN – IgA vasculitis without nephritis, IgAVN – IgA vasculitis nephritis, ESR – erythrocyte sedimentation rate, CRP – C reactive protein, Hb – hemoglobin, L – leukocytes, Plt – platelets, BUN - blood urea nitrogen, E/mm3 – erythrocytes per mm3 of urine, 24h protein ex.- 24h urinary protein excretion, eGFR – estimated glomerular filtration rate

Table S2. Routine laboratory findings in patients 6 months since the disease onset (Mann–Whitney U test)

	group	N	Min.	Max.	Centile			P
					25.	Median	75.	
ESR (mm/h)	IgAVwN	55	2.00	30.00	4.00	7.00	11.00	0.887
	IgAVN	42	1.00	23.00	4.75	7.50	11.25	
CRP (mg/L)	IgAVwN	55	0.10	34.10	0.30	0.30	1.00	0.896
	IgAVN	42	0.10	19.90	0.30	0.30	0.93	
Hb (g/L)	IgAVwN	55	108.00	147.00	122.00	129.00	136.00	0.771
	IgAVN	42	109.00	156.00	123.75	129.50	136.25	
L (x 10^9/L)	IgAVwN	55	2.60	11.90	7.00	8.10	9.20	0.667
	IgAVN	42	4.71	11.70	6.74	7.70	9.33	
Plt (x 10^9/L)	IgAVwN	55	176.00	503.00	267.00	311.00	378.00	0.665
	IgAVN	42	206.00	472.00	268.50	323.50	376.50	
BUN (mmol/L)	IgAVwN	55	2.30	7.80	3.30	3.90	5.00	0.345
	IgAVN	42	2.20	6.10	3.20	3.84	4.33	
Creatiin (mmol/L)	IgAVwN	55	28.00	65.00	35.00	41.00	47.00	0.010
	IgAVN	42	27.00	78.00	39.75	45.50	55.50	
Tot. proteins (g/L)	IgAVwN	55	6.27	14.29	8.20	9.54	10.80	0.489
	IgAVN	42	4.82	13.52	8.12	9.03	10.37	
IgA (g/L)	IgAVwN	55	0.66	2.95	1.10	1.52	1.79	0.164
	IgAVN	42	0.79	4.12	1.34	1.60	1.90	
IgM (g/L)	IgAVwN	55	0.38	1.97	0.68	0.84	1.01	0.067
	IgAVN	42	0.47	1.80	0.74	1.05	1.23	
IgG (g/L)	IgAVwN	55	6.27	14.29	8.20	9.54	10.80	0.489
	IgAVN	42	4.82	13.52	8.12	9.03	10.37	
E/mm^3	IgAVwN	55	0.00	5.00	0.00	0.00	0.00	< 0.001
	IgAVN	42	0.00	8553.00	0.00	4.00	15.25	
24h protein ex.	IgAVwN	55	0.02	0.16	0.05	0.07	0.09	0.080
	IgAVN	42	0.03	0.85	0.06	0.08	0.09	
eGFR (ml/min/1,73m^2)	IgAVwN	55	78.86	154.56	97.64	110.93	125.50	0.006
	IgAVN	42	67.40	146.04	88.61	100.17	114.55	

Legend: IgAVwN – IgA vasculitis without nephritis, IgAVN – IgA vasculitis nephritis, ESR – erythrocyte sedimentation rate, CRP – C reactive protein, Hb – hemoglobin, L – leukocytes, Plt – platelets, BUN - blood urea nitrogen, E/mm3 – erythrocytes per mm3 of urine, 24h protein ex.- 24h urinary protein excretion, eGFR – estimated glomerular filtration rate

Table S3. Correlation of e-GST and standardly used marker of kidney function impairment in patients with IgAV

		IgAVN (N=42)			IgAVwN (N=55)		
		e-GST 1	e-GST 2	e-GST 3	e-GST 1	e-GST 2	e-GST 3
eGFR 1	Correlation Coefficient	-0.001			0.137		
	P	0.996			0.318		
eGFR 2	Correlation Coefficient		0.135			0.002	
	P		0.394			0.986	
eGFR 3	Correlation Coefficient			-0.151			0.129
	P			0.340			0.348
E/mm^3 1	Correlation Coefficient	0.138			-0.300		
	P	0.383			0.026		
E/mm^3 2	Correlation Coefficient		0.569			-0.071	
	P		**<0.001**			0.608	
E/mm^3 3	Correlation Coefficient			0.698			0.098
	P			**<0.001**			0.476
24h ex. 1	Correlation Coefficient	0.262			0.226		
	P	0.094			0.098		
24h ex. 2	Correlation Coefficient		0.227			-0.161	
	P		0.148			0.241	
24h ex. 3	Correlation Coefficient			0.442			0.074
	P			0.003			0.590
Creat. 1	Correlation Coefficient	-0,038			0,024		
	P	0,809			0,865		
Creat. 2	Correlation Coefficient		0,282			0,149	
	P		0,070			0,276	
Creat. 3	Correlation Coefficient			0,281			0,114
	P			0,071			0,407

Legend: IgAVwN – IgA vasculitis without nephritis, IgAVN – IgA vasculitis nephritis, e-GST – erythrocyte glutathione S-transferase activity, eGFR – estimated glomerular filtration rate, E/mm3 – erythrocites per mm3 of urine, 24 h ex.- 24 h urinary protein excretion, Creat. - Serum creatinine

Table S4. Correlation of e-GST activity between patients with IgAVN with initial normal and abnormal urine findings

IgAVN	e-GST	N	Min.	Max.	Centile			P
					25.	Median	75.	
initial normal urine findings	e-GST 1	8	4.20	7.00	4.20	4.30	5.05	0.053
initial abnormal urine findings		34	3.40	10.02	4.75	5.80	7.50	
initial normal urine findings	e-GST 2	8	4.50	7.80	5.55	6.10	6.95	0.152
initial abnormal urine findings		34	1.50	9.60	4.1	4.80	6.05	
initial normal urine findings	e-GST 3	8	4.10	6.20	4.45	5.30	5.75	0.392
initial abnormal urine findings		34	1.20	9.50	3.10	3.80	5.85	

Legend: IgAVN – IgA vasculitis nephritis, e-GST – erythrocyte glutathione S-transferase activity
